# The impact of environmental and social sustainability on the reshoring decision making and implementation process: insights from the bicycle industry

**DOI:** 10.1007/s12063-023-00372-1

**Published:** 2023-05-22

**Authors:** Luciano Fratocchi, Julia Mayer

**Affiliations:** 1 Department of Industrial and Information Engineering and Economics, University of L’Aquila, L’Aquila, Italy; 2 Copernicus Institute of Sustainable Development - Section Innovation Studies, Utrecht University, Utrecht, The Netherlands

**Keywords:** Rehoring, Backshoring, Bike industry, Offshoring, Sustainability

## Abstract

After decades of huge production offshoring, companies are increasingly re-evaluating their production footprint, often implementing so-called reshoring strategies. Among them scarce attention has been devoted to the near-shoring option, i.e., relocation to the home region. At the same time, the impact of environmental and social sustainability on such strategies is an emerging issue within the reshoring scholars’ debate. This paper aims to shed new light on this debate focusing on the bike industry. To reach the research aim, a single case study was investigated, regarding an Austrian bike manufacturer that decided to near-shore the assembling phase to Poland in 2021. Collected evidence was analyzed through an interpretative framework based on the extant literature, allowing us to understand the impact of environmental and social issues on the reshoring decision making and implementation process, and its outcomes. The analyzed case study shows that environmental and social issues may play different roles when near-shoring decisions are taken and implemented. However, it emerges that the magnitude of such impacts may differ among the specific levels of analysis investigated (namely drivers, barriers, enabling factors and outcomes) and the sustainability pillar investigated (environmental vs. social one). The debate on sustainability impacts on a firm’s relocation strategies is still in its infancy, moreover the near-shoring alternative was not considered earlier in the academic debate. Therefore, this paper is the first attempt to shed new light on this issue and also proposes some future research avenues.

## Introduction

After decades of huge manufacturing offshoring (Podrecca et al. 2021; Kinkel and Maloca 2009; Kinkel 2012), generally due to efficiency-seeking aims, in recent years companies have started to review their previous offshoring decisions by implementing so-called reshoring strategies, also referred to as “relocations of second degree” (Barbieri et al. 2019). More specifically, some manufacturing companies decided to relocate earlier offshored production activities to the home country (back-shoring), the home region (near-shoring), or a country far away (further offshoring) (Fratocchi et al. 2014). Even if the term reshoring has been adopted as a synonym for specific relocation to the home country, in this paper we will refer to it as a generic relocation of second degree.

When analyzing the extant reshoring literature, it clearly emerges that while back-shoring has been attracting a huge amount of interest by scholars (Barbieri et al. 2018; Merino et al. 2021; Stentoft et al. 2016; Wiesmann et al. 2017), the other two alternatives have rarely been addressed in the literature (e.g., Mohiuddin et al. 2019; Piatanesi and Arauzo-Carod 2019). It is worth noting that Ancarani et al. (2015) consider back- and near-shoring as similar strategies. At the same time, Piatanesi and Arauzo-Carod pointed out that near-shoring “is still a limited phenomenon […] but it is reasonable to assume [… it …] will relatively soon increase significantly”, since it combines the advantages of both offshoring and back-shoring strategies (2019, 818). Finally, Merino et al. (2021) recently suggested further investigations should be undertaken to better understand the specificities of near-shoring strategies.

Moreover, it is widely recognized that production activities affect both the environmental and social pillars of a firm’s sustainability performance. Consequently, decisions regarding where companies manufacture their products have a tremendous impact on such pillars (Chen et al 2014; Sarkis and Zhu 2018). However, when analyzing the reshoring extant literature, it clearly emerges that the role of environmental and social sustainability has rarely been investigated, even if some authors explicitly called for contributions on this issue (Fratocchi and Di Stefano 2019; Orzes and Sarkis 2019).

Based on the previously described evidence, this paper aims to shed new light on the near-shoring phenomenon, specifically focusing on the impacts (if any) of environmental and social sustainability on the location decision-making and implementation process and its outcomes. In so doing, we integrate the comprehensive framework developed by Boffelli and Johansson (2020) with findings by Fratocchi and Di Stefano (2019), which specifically focus on sustainability issues within reshoring strategies. Based on the similarity between near- and back-shoring alternatives earlier claimed by Ancarani et al. (2015), we assume both frameworks may also be easily applied to the decision to relocate production activities to the home region, instead of the home country. In this respect, while Boffelli and Johansson’s (2020) approach includes both offshoring and reshoring strategies, dividing each into three main steps (namely, decision-making, implementation, and outcomes), Fratocchi and Di Stefano (2019) postulates sustainability may act as a motivation/driver of the relocation decision and/or an outcome, barrier and/or enabling factor to its implementation. The integration of these two frameworks allows us to deeply investigate case studies regarding relocation decisions since it permits analysis of the entire process and its outcomes. Based on this integrated framework, we aim to investigate the following research questions:

RQ1: How do environmental and social sustainability issues impact the near-shoring decision-making process?

RQ2: How do environmental and social sustainability issues impact the near-shoring implementation process?

RQ3: What are outcomes related to environmental and social sustainability resulting from the near-shoring relocation process?

To reach this research aim, a single case study regarding Woom, an Austrian bike manufacturer for children and teenagers was developed.

The rest of the paper is organized as follows. In the first section, the extant literature addressing the relationship between environmental and social sustainability and relocations of second degree is summarized and the interpretative framework is presented. In the next section, methodological issues are addressed, explaining the preference for the single case study approach and the procedure adopted to collect data and to analyze them. The third section is then devoted to a description of the investigated company, while the fourth one summarizes collected evidence related to the impact of sustainability on the three steps of the near-shoring process, namely decision-making, implementation and outcomes. After this, the discussion is presented in the fifth section, which is then followed by conclusions, implications for scholars, managers and policy-makers, and limitations.

## Theoretical framework

As earlier noted, literature on near-shoring strategies is still in its infancy. However, Ancarani et al. (2015) consider back- and near-shoring as similar strategies. Therefore, we assume that findings proposed in the back-shoring extant literature may also be useful to define a theoretical framework for the near-shoring alternative. In order to develop one that is useful to support our research aims, the reshoring extant literature was carefully analyzed. Firstly, we focused on the few articles addressing the impact of sustainability issues on the relocations of second degree. Secondly, we analyzed publications describing the decision-making and implementation process adopted in the case of relocations of second degree. Based on them, we defined a theoretical framework combining the phases of reshoring strategy (decision making, implementation and outcomes) and the possible impacts of environmental and social sustainability. Based on such a framework, in the next section we will investigate the chosen reshoring case study.

### Sustainability issues in relocation of second degree

Within the extant literature regarding relocations of second degree, environmental and social sustainability issues have rarely been addressed, despite the positive impact such decisions may have in terms of reduction of CO2 emissions (for products addressing the home and regional markets) and the adoption of fairer working conditions generally adopted in Western countries when compared to Asian ones (Fratocchi and Di Stefano 2019; Orzes and Sarkis 2019). In this respect, Orzes and Sarkis (2019) pointed out that the one between sustainability issues and reshoring is a “foundational unexplored relationship” (p. 482) that should be investigated according to a systematic approach. At the same time, in their structured literature review, Fratocchi and Di Stefano (2019) identified only 28 Scopus indexed documents generically referring to environmental sustainability and 25 to social ones; finally, 22 of them simultaneously addressed both issues.

More recently, Pourhejazy and Ashby (2021) pointed out that environmental and social sustainability issues should be carefully assessed when evaluating whether to relocate production activities, since they may have a long-term impact on the cost-efficiency of the post-reshoring decisions. Finally, Choudhary et al. (2022) pointed out that “sustainable supplier selection and reshoring strategy can and should be pursued mutually” (Choudhary et al. 2022, 14).

Of special note is the role of process innovations adopted while implementing reshoring strategies. Martínez-Mora and Merino (2020) showed how the emergence of a new eco-friendly technology in the jeans business allowed several companies to reshore the dyeing production phase to their home country. At the same time, the recent study from Cosimato and Vona (2021) points out the relevance of eco-friendly innovations and the adoption of production automated processes while implementing reshoring strategies.

Very few authors specifically collected empirical evidence of reshoring strategies where sustainability issues assumed a relevant role. Among them, Ashby (2016) proposed a longitudinal analysis of a UK company that succeeded in reconstructing a ‘100% made-in UK’ supply chain in the garment industry, greatly reducing the carbon footprint of its products. At the same time, Fratocchi and Di Stefano (2019) analyzed the case of TES Scandinavia, a producer of air treatment systems pushed to relocate and re-insource from Canada following the European Union (EU) ban on fluorinated greenhouse gasses. The company decided to redesign the product making it more eco-friendly; moreover, it built a completely local supply chain and employed people who had been fired by a multinational company that had decided to offshore production activities. More recently, Moradlou et al. (2020) investigated the case of a UK laundry detergent manufacturer that, after back-shoring production, developed eco-friendly products, more consistent with its marketing orientation. However, all the sampled case studies regard the back-shoring alternative, while no previous study offers specific insights on the near-shoring one.

When considering the possible role of sustainability within the reshoring decision-making process, Orzes and Sarkis (2019) hypothesize that environmental and social sustainability may act either as a reshoring motivation or an enabler/facilitator. In this respect, Fratocchi and Di Stefano (2019) pointed out that these two pillars of sustainability may also act as either a barrier or an outcome of the implemented relocation strategy. Moreover, the authors showed that, while all the sampled contributions conceptualized sustainability as a motivation, very few of them specifically referred to the barrier, enabler, and outcome perspectives.

For the purpose of this article, the three elements proposed by Fratocchi and Di Stefano (2019) will be adopted to investigate the selected reshoring company.

### The reshoring decision-making and implementation process

When examining the existing reshoring literature, it emerges that nine Scopus indexed journal articles specifically address the decision-making and implementation processes related to relocation of second degree, namely Bals et al. 2016; Benstead et al. 2017; Boffelli and Johansson 2020; Boffelli et al. 2018, 2020; Gray et al. 2017; Joubioux and Vanpoucke 2016; Oshri et al. 2017; Schmidt et al. 2017. Seven of them contained frameworks for only the back-shoring strategies (Bals et al. 2016; Benstead et al. 2017; Boffelli and Johansson 2020; Boffelli et al. 2018, 2020; Gray et al. 2017; Oshri et al. 2017); one considered further offshoring alternatives and options to remain offshore (Joubioux and Vanpoucke 2016), and the last one refers to general alternatives to the international location of business activities (Schmidt et al. 2017). The framework by Gray et al. (2017) specifically addressed small and medium-sized enterprises, while the approaches of the others were more general. The proposal by Bals et al. (2016) was based on a literature review only, whereas the others were also practically validated using case studies (Benstead et al. 2017; Boffelli and Johansson 2020; Boffelli et al. 2018, 2020; Gray et al. 2017; Joubioux and Vanpoucke 2016) or surveys (Oshri et al. 2017; Schmidt et al. 2017).

**Fig. 1:**
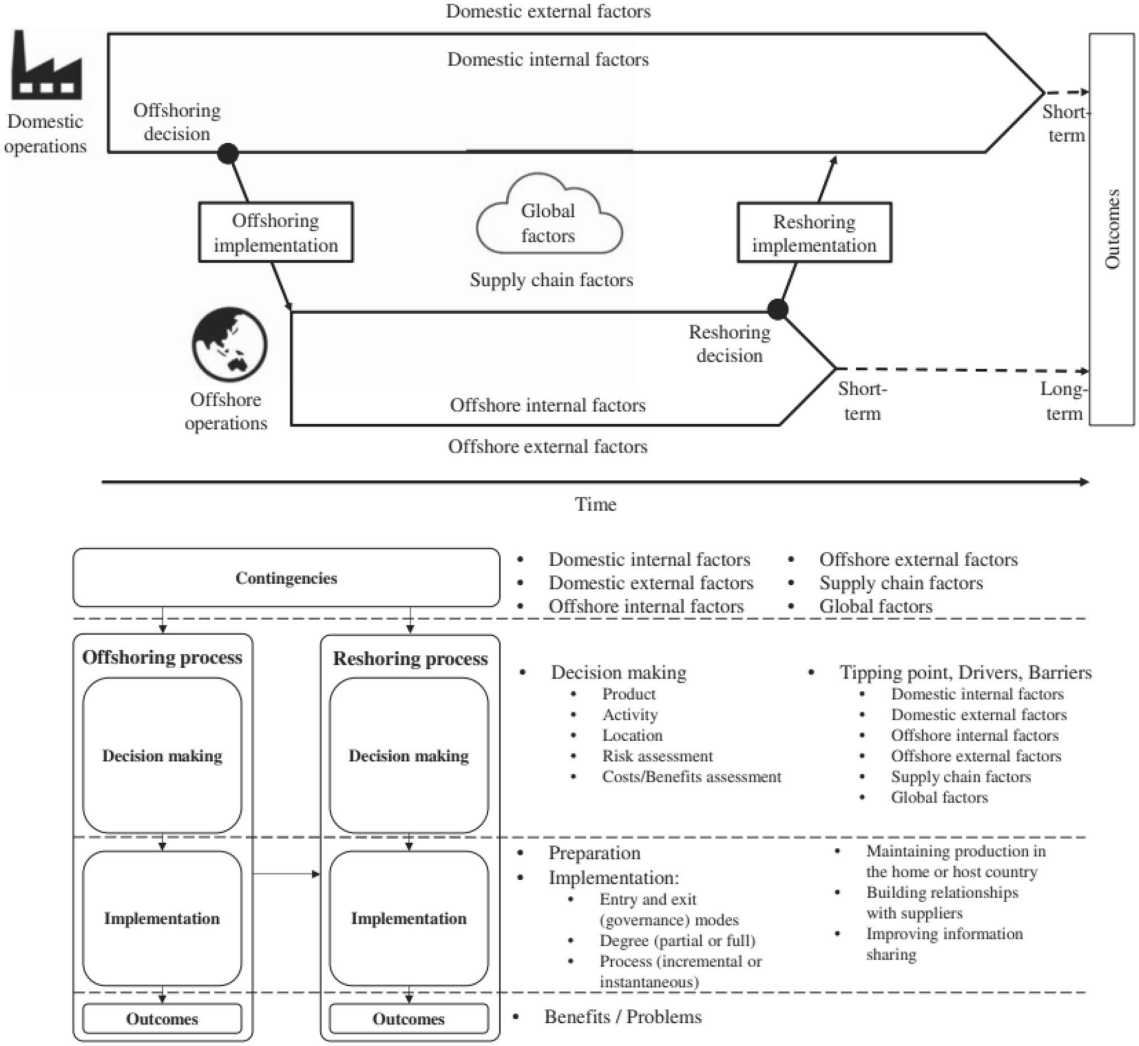
The Boffelli and Johansson comprehensive theoretical model Source: Boffelli and Johansson 2020

Among the selected frameworks, the one proposed by Boffelli and Johansson (2020) is the most comprehensive one, since it includes both the offshoring and reshoring processes (Fig. 1). However, for the purpose of our paper, attention will be focused only on the reshoring one. The contribution of the framework under discussion for the purpose of this paper is manifold:

- Firstly, Boffelli and Johansson (2020) suggest the reshoring process is influenced by several contingencies, including internal (firm level) and external factors (e.g., supply chain and global factors). Based on such an assumption, the selection of the case company should be carefully conducted considering also the characteristics of its industry in terms of reshoring decisions and sustainability strategies;
- Secondly, the authors suggest analyzing reshoring case studies at three different levels, namely Decision making, Implementation and Outcomes. Therefore, the analysis of the impact of sustainability issues should be replied at each of these three levels. In this respect, it is worth noting that Boffelli and Johansson (2020) conceptualize outcomes as either positive or negative effect of the entire reshoring process, while Fratocchi and Di Stefano (2019) consider only the one related to environmental and social sustainability;
- Thirdly, the framework explicitly refers to drivers and barriers, that are two of the dimensions suggested by Fratocchi and Di Stefano (2019) as possible impacts of sustainability on the reshoring process.

### The proposed integrated theoretical framework

Based on the earlier review of the extant reshoring literature, it is possible to design an integrated theoretical framework combining the Boffelli and Johansson (2020) and the Fratocchi and Di Stefano (2019) frameworks. As earlier pointed out, for the purpose of this paper the Boffelli and Johansson (2020) one is considered only with respect to the reshoring process. This allows us to integrate it with the Fratocchi and Di Stefano (2019) framework which was proposed to analyze the impact of sustainability issues in the case of relocations at the home country. In order to have a more detailed analysis, it is suggested to divide the analysis of Outcomes into Benefits and Problems, distinguishing between short and medium/long term ones. At the same time, following Fratocchi and Di Stefano (2019), the role of environmental and social sustainability should be investigated at each level in terms of driver, barriers, enabling factors and sustainability related outcomes. However, the three issues have not the same relevance for each of the three levels. More specifically, drivers are generally recognized as relevant for the decision making phase, since they motivate the firm’s decision to relocate its manufacturing activities (Fratocchi et al. 2016). At the same time, enabling factors characterize only the implementation phase. A special note deserves the role of barriers. While in the Boffelli and Johansson (2020) framework they are included only in the decision making step, they could have an impact also in the implementation phase, as pointed out by Boffelli et al. (2021). Finally, as in Boffelli and Johansson (2020), contingencies characterize and influence all the reshoring process.

**Fig. 2:**
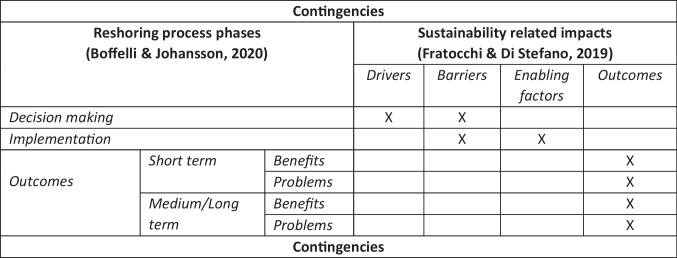
The proposed integrated framework

The integrated theoretical framework proposed to investigate the role of (environmental and social) sustainability on the reshoring process is then based on three distinct levels of analysis (Decision making, Implementation and Outcomes) and four types of impacts (namely Drivers, Barriers, Enabling factors and Outcomes) for each of the two sustainability pillars (environmental and social ones). The framework is summarized in Fig. 2.

## Methodology

As earlier stated, the paper aims to investigate the role (if any) of sustainability (environmental and social pillars) within the decision-making, implementation process and outcomes of a near-shoring strategy. Due to the novelty of the topic, a qualitative research method has been chosen (Bryman 2012) focusing on a single case study (Yin 2003). More specifically, the unit of analysis is the near-shoring decision-making, implementation process, and the outcomes of this decision.

The case was selected after applying a purposive sampling approach, meaning that the case selected was expected to best provide the information needed to answer the research questions successfully with the help of predefined criteria to increase the study’s reliability (Bryman 2012; Etikan et al. 2016). Additionally, Bernhard (2011) stresses purposive sampling is used, especially when studying “intensive case studies” (p. 698). Since the focus of the firm-level analysis was to conduct a thorough analysis of the aspects influencing each step of the decision-making and implementation process, an intensive case study approach was selected. The case selection was based on the following criteria:

- First of all, the company should belong to an industry characterized by heavy offshoring waves in the past decades but also by a growing number of relocations at the home country and/or region in a more recent period. In this respect, the bike industry emerged as an adequate alternative, since it has been characterized by huge offshoring waves since the 1960s, when the Taiwanese government promoted an export-led economic growth mainly focused on bikes (Ferry 2021). At the same time, in 1990 China started to subsidize the national industry in order to become the world’s factory for bikes. To sum up, around 50% of bikes exported at the worldwide level comes from either China or other Asian countries; the figures are even larger when you refer to bike components. However, European bike makers have been implementing relocations at least since 2018 at a growing rate and further waves are expected in 2022–2023 (Banca IFIS 2022). In this respect, it is worth noting that some European countries are developing so-called “bike valleys” to attract relocation of bike production (e.g., Portugal, which became the first European country by number of produced bikes (e-bikes excluded); Beckendorff 2021). A special note deserves Eastern European countries which are attracting huge production investments by both European and Asian companies (Burgin 2022). Moreover, several companies operating in the bike industry signed the “Shift Climate Commitment”, which “aims to drive positive climate actions across the industry” (www.shiftcyclingculture.com/climatecommitment). Based on such an agreement companies are committed to measure every year their greenhouse gas emissions and to reduce them at least 55% by 2030. At the same time, some German and Austrian companies operating in the bike industry also signed the “Bike Charta”, “an action plan for corporate social responsibility, resource conservation and climate protection” (www.bikebrainpool.de). Finally, the bike is generally conceptualized as a product with a potential to make the world a little bit better supporting a more sustainable mobility. In this respect, the Covid-19 pandemic boosted the bike demand in Europe; however, due to the almost saturated production capacity of Asian manufacturers, customers were requested to wait for a longer time, sometimes even 6–12 months until their bicycles were delivered (Buehler and Pucher 2021). Therefore, several companies either already decided or are deciding to relocate production activities to the home region/country to gain in terms of responsiveness to customers’ request;
- Secondly, the relocation of the production activity should have, at least potentially, effects on sustainability issues. In this respect, the wide offshoring of bike production in East Asia implies a huge level of CO2 emissions for all products marketed on the European market. Moreover, the possibility to reduce production and delivery time to the final market allows European companies to match the dramatically growing demand for bike and, in turns, the development of more sustainable mobility practices in European countries;
- Finally, the company should have already implemented the near-shoring relocation strategy, otherwise it would be impossible to analyze the three steps of the reshoring process. As far as the outcomes, at least the short term ones should be reached.

In order to choose the reshoring case study within the bike industry, the UnivAQ Manufacturing Reshoring Dataset (Fratocchi and Di Stefano 2020) was checked. The dataset extends the previous Uni-CLUB MoRe reshoring one (Ancarani et al. 2015; Wan et al. 2019) and includes evidence by the European Reshoring Monitor dataset (Ancarani et al 2019; Barbieri et al. 2019; Eurofound 2019). The UnivAQ Manufacturing Reshoring Dataset contains information on more than 1400 manufacturing reshoring decisions, gathered from several sources; for each observation, the recorded information includes: the name of the company involved; company size; industry; country of origin; year in which reshoring was implemented; “abandoned” host country; and declared motivations. A total number of seven bike manufacturers were found and each of them was contacted to verify their collaboration. The Austrian bike maker Woom, producing bikes for children, agreed to participate in the research. Since its establishment in 2013, the company offshored and outsourced – at least partially – the production processes, first of all to a Czech contractor and then it further offshored to Asian countries. However, at the beginning of 2021, it near-shored part of the assembling activity to Poland.

Data were collected from semi-structured interviews conducted with seven key informants, two of which are on the board of management; the other five belong to the following departments: supply chain management, quality assurance, marketing, communication & public relations, corporate social responsibility. After specifying these departments which we liked to be involved in the study, our contact person from Woom chose the interviewees in coordination with us. The same questions were asked to all interviewees (see Appendix A), however, the level of expertise per interviewee varied and, therefore, also the length and detail of each response.

The interviews were held online via MS Teams and lasted between 30 and 80 min. Each interviewee was given the opportunity at the end of the interview to comment or still change their answers. For the analysis, the interviews were recorded, transcribed, and then coded. To keep an overview of the answers of the different respondents, they were given numbers from 1–7, which is also indicated behind direct quotations in the result section. More specifically, coding was conducted through a data driven open-coding approach (Gibbs 2007). The authors autonomously conducted the coding activity following the dimensions of the proposed theoretical framework (sustainability pillars, levels of analysis, and typology of impacts. After comparing the coded output, no relevant differences emerged. After the analysis the draft of the paper was sent to the company to give them the possibility to comment and review the results and for receiving their final approval.

## The case study company: woom GmbH

### Company description

Marcus Ihlenfeld and Christian Bezdeka define themselves as “bike-mad dads” aiming “to develop a bike for their kids that would perfectly fit the proportions and riding behavior of children” (woom.com). They have complementary skills; more specifically, Christian is a trained biomedical technician with a degree in industrial design and years of experience within the bike industry, while Marcus is a marketing manager with an extensive practice in the automotive industry. Their business idea became reality in 2013 when the production of the first 287 bikes started in Marcus’ garage in Vienna. Since then, the company’s mission has been “to create products of such exceptional quality that they provide children and their parents with unforgettable experiences. Our hope is that children who experience these magical moments on their bikes will grow up to be adults who regularly ride their bikes and become ambassadors of the transport revolution” (woom.com). At the same time, the entrepreneurs summarize their vision as follows: “We see cycling as a powerful force for making the shift to climate-friendly, healthy, efficient, and enjoyable urban and rural mobility. Every single Woom bike brings the world one step closer to sustainable travel and a more livable future” (woom.com).

Based on such vision and mission, the founders focused their strategy on the production of premium ultralight children's and teenagers' bikes. Today, the company offers three different product lines:

- Woom Original: it includes seven models of all-rounder superlight bikes for children aged between 18 months and 14 years. Price ranges between € 189 and € 539;
- Woom Off & Off Air: includes ultralight (from 7, 8 to 11 kg) but robust “off-road buddies”, with and without front suspension, for children aged six and up. The price of the six models (three for each brand), ranges between € 749 and € 999;
- Woom Up: includes superlight premium electric mountain bikes with a child-friendly drive system for riders aged from seven up to 14. Prices of the two models range from € 2,890 to € 2,990.

All the Woom products share some common features:

- smart engineering to meet the needs of children;
- lightweight, up to 40% less than other children’s bikes;
- high-value retention in the used-bike market (this issue is quite relevant, considering that children grow fast, therefore they generally use the same bike for no more than three years);
- high safety and comfort levels;
- timeless design due to sleek silhouettes and clean lines.

The company has promoted its products in foreign markets since its founding: in 2014, Marcus’ brother founded Woom USA and, by the end of 2016, the company’s products were sold in around 10 countries. In the same year, the company participated for the first time at Eurobike, Europe’s most important fair for the bike industry. Two years later, the company won several prizes for its product designs and sold around 80,000 bicycles in a year. In 2019, the company enlarged its product portfolio by launching the first mountain bike (Woom off) and electric mountain bike (Woom UP) and presenting its own clothing line. Now, the company is represented in 30 countries in Europe, Asia, and North America. Moreover, it is the market leader in Germany, Austria, and Switzerland; finally, 80% of sales come from Europe.

Table 1 summarizes sales growth since the Woom establishment.

**Table 1:** Woom’s sales (number of bicycles)

Year	Bikes
2013	287
2014	1.287
2015	4.772
2016	12.133
2017	45.936
2018	79.754
2019	141.115
2020	228.000
2021	287.500

Source: Firm’s data (woom.com)

More than 150 employees operate in Austria and around 40 in the USA; however, it is expected this will reach 300 people by the middle of 2022.

### The role of sustainability in the Woom strategy

Woom addresses sustainability in its business model and has formulated commitments and strategies, and implemented several initiatives that contribute to making its business more sustainable. As already pointed out, the company’s mission is to positively influence and inspire children by designing and marketing its high-quality bicycles in such a way that children enjoy and use this means of transport continuously and in the future.

In terms of environmental sustainability, the company signed the “Shift Climate Commitment” based on which it is committed to slash its CO2 emissions by 2030.

To underline its ambitions, the company has committed itself to Sustainable Development Goals (SDGs) and established a corporate social responsibility (CSR) team to monitor and develop ways to contribute to the SDGs successfully. For instance, in 2019 Woom collaborated with an NGO ‘World Bicycle Relief’ and started a fundraiser campaign called ‘Bells for a Better Life’ to increase mobility and independence in rural areas in countries of the Global South by providing bicycles to the local population in these regions (woom.com).

It is worth noting that sustainability is also a relevant issue in Woom’s supply chain strategy since the company requests from its production partners the same commitment to ethical, social, and environmental responsibility. More specifically, contractors involved in the production of accessories and clothing are required to sign a detailed Code of Conduct. At the same time, the company aims to further expand this agreement to other suppliers, including the manufacturers of bicycle components (woom.com). In this respect, Woom joined the Bike Charta developed by a set of German and Austrian companies operating in the bike industry. This agreement includes rules regarding relationships with suppliers in order to reduce the environmental impacts of their products and services. Moreover, special attention is given to the working conditions adopted in the contractors’ factories, where the company conducts specific audits regularly. *Evolution of the production footprint.*

When considering production activities, Woom may be defined as a selective “born offshored” company (Moradlou et al. 2020), since the founders decided to offshore the production of its frames shortly after the company’s establishment. More specifically, they established a partnership with the Czech company Fort Frames; the company was founded by Miroslav Samek in 1992 and is located in Ústí Nad Orlicí, around 200 km from Prague in the East. It specializes in the production of small lots of highly customized frames developed in cooperation with the customer (bike-eu.com). This business model was consistent with the one from Woom since sales were still limited (compared to other already established competitors). Moreover, Woom requested highly customized frames, since Christian had developed “his own size tables to perfectly adapt the bikes to the needs and proportions of children” (woom.com) having found it impossible to find this type of data online. Frames produced by the Czech partner were then assembled by Woom in Austria.

However, when production volumes increased, Woom was no longer satisfied with the quality of the frames supplied by the Czech partner; the supplied frames were not always identical, thus the production was not scalable. Moreover, the growing request for Woom products (from 287 in 2014 to 5,000 in 2015) suddenly made in-house assembling no longer affordable. Finally, the production capacity available at the Czech partner rapidly became too limited. Therefore, in late 2017, the two founders decided to establish a collaboration with a Cambodian company, Asama Speedtech which could manage the entire production process. This company is a subsidiary of the Taiwanese Asama Group, one of the world’s largest bike manufacturers producing up to one million bikes per year for the North American, Western European, and Asian markets. It employs more than 4,000 people and owns three factories in Taiwan, Cambodia, and Vietnam. The Group is vertically integrated since the Vietnamese subsidiary also produces bike parts and components (https://asama-bike.com). The choice of a partner located in South-East Asia was justified by the huge offshoring waves of bike production activities to Taiwan and other countries in the region since the ‘70 s. Therefore, in 2017 most of the latest technical know-how was located in this area. Moreover, the production in Asia allowed Woom to directly serve the local market, which rapidly assumed a certain relevance for the company. Finally, it was also a useful logistics base from which to export bikes to the US market, where the company had opened a warehouse in Texas.

The positive results obtained by collaborating with the Cambodian partner and the further growing demand coming from the worldwide market (80.000 bikes in 2018 and around 145.000 in 2019), induced the company to search for additional Asian partners. Therefore, in 2020 collaborations were initiated with the Bangladeshi M&U and the Vietnamese Asama Qualitech. While the latter is the local subsidiary of the Asama Group, the former belongs to the Meghna Group, one of the largest conglomerates in Bangladesh. In the last years, Bangladesh M&U reached a total production of 1.3 million bikes which were exported to several foreign countries (Ahosan Habib 2020).

However, due to the great relevance of the European market (around 80% of total sales) for Woom, in 2019 the company decided to partially relocate the production they had offshored in Asia. It is worth noting that Woom’s managers recognized an increasing constraint in the production capacity of Asian contractors, due to the fast-growing request for bikes at the global level. One of the interviewees described the situation as follows: *“before they [Woom’s contractors in Asia] got really constrained, we had already made our decision to say, we are now looking for a factory closer to Austria where we can also transfer methods and develop know-how more easily”* (IW_1). At the same time, the fast-growing demand for Woom’s products allowed the company to contact large bike manufacturers located in Europe (mainly Eastern European countries). In this respect, one of our respondents pointed out “*a few years ago, when we sold only a few thousand bicycles, it was difficult to partner up with large manufacturers. At this point, we move quantities that open doors—even to large producers”* (IW_ 3). However, such bargaining power was not available compared to Asian partners since their production capacities were higher than that of European bike makers. More specifically, one of the respondents noted *“they [Asian contractors] are partially already doing what they want with us. To a certain extent, they dictate delivery conditions and prizes”* (IW_3). By moving the production partially to Europe the company aspired to gain part of its independence in the manufacturing process and continuously shape and develop its supply chain according to its future growth objectives. At that time, the option to in-source immediately emerged as not viable since the know-how necessary to internally produce bikes was completely missing. More specifically, one of the company's respondents clearly stated: *“We can develop bicycles. We know exactly what we want to manufacture. But that doesn't necessarily mean that you're the best at manufacturing it”* (IW_3).

Therefore, the company started to select European-based manufacturers. In so doing Woom managers met the German Sprick Cycle GmbH, a family-owned company that produced in Germany until 2004 and then offshored to Poland where they established an assembling plant (https://sprick-cycle.de). Concerning the selection process of this supplier, one of the interviewees at Woom clearly stated *“You don't have much of a choice. There are only a handful of companies in Europe that can build bicycles the way we want and need them. And there you can't choose the country; you have to be happy when you find someone who builds it. In our case it was Poland. Romania could have been an option, and there are companies in Portugal”* (IW_3). However, *“it is also important that the partner can deliver the quality that we demand”* (IW_3). Therefore, the German partner emerged as an excellent option.

By February 2021, two production lines and two highly automated spoke machines were activated in the Polish plant employing 80 of the 300 people working there. At the moment, the production relocation is regarding only the Woom Original product line, addressed to children aged between 18 months and 14 years. However, further production lines for assembling are under construction to realize the Woom OFF mountain bikes. It was expected that the German partner would assemble up to 115.000 bikes by the end of 2021, which is around 40% of the total annual production. However, the firm’s ultimate goal is to supply the European market with bikes made in Europe. In contrast, the Asian partners’ production will serve the local market and the North American one. Therefore, a new collaboration with the Cambodian Evergrand will be enacted by 2022 (www.woom.com).

In Table 2 the evolution of the Woom production footprint is summarized.

**Table 2:** The evolution of Woom production footprint

Year	2013–2016	2017–2019	2020	2021	2022
Location strategy	Off-shoring (partial)	Off-shoring (total)	Further off-shoring	Near-shoring (partial)	Further off-shoring
Production activity	Frame Production	Production & Assembling	Production & Assembling	Assembling	Production & Assembling
Host country	Czech Republic	Cambodia	Bangladesh & Vietnam	Poland	Cambodia

## The Woom near-shoring strategy and the role(s) of sustainability issues

### The decision-making phase

When analyzing the Woom near-shoring decision-making phase according to the proposed integrated theoretical framework, two key issues should be carefully investigated, namely the motivation/drivers and the barriers. While our focus is on (environmental and social) sustainability issues, in order to have a wider understanding of the sampled reshoring process, in this section attention will be devoted to all the motivations/drivers and barriers emerging in this first phase. The sustainability issues will be discussed in more detail in the next section.

Based on the collected interviews with the seven respondents, 14 different drivers were cited as relevant for the company’s decision to near-shore (Table 3). They will be described and discussed in the following, paying specific attention to the ones related to sustainability issues.

**Table 3:** Motivations influencing the near-shoring decision

Drivers	Number of respondents (up to 7)
Vicinity to European customers	5
Company’s sustainability values	4
Better environmental sustainability performance (e.g. CO2 emissions)	4
Minimization of transport	3
Shorter lead times	3
Risk minimization	3
More control over production activities	3
Availability of Asian contractors in terms of production capacity and technical collaboration	3
Independence from Asian producers	2
Better brand image	2
Increasing customer satisfaction	1
Reliability in production of the European Partner	1
Lower working capital	1
Increased flexibility	1

As pointed out by one of the respondents, “*economic factors were paramount*” (IW_ 6), especially those related to operation, supply chain management and marketing issues. In this respect, the possibility of being closer to Woom’s European customers was cited by five out of the seven respondents. More specifically, one of the interviewees emphasized the following: *“It takes two years from the moment we order a bike until it gets delivered. Obviously, having a production closer at hand is a great thing*” (IW_5). More specifically, the interviewee refers to the time span requested by Asian suppliers to make the bike available to Woom. Such data are even larger than the ones earlier reported by Gylling et al. (2015) (six months) due to the combined effect of the growing demand for bicycles (especially in Europe, during the Covid-19 pandemic; and the high degree of saturation of the production capacities of Asian manufacturers (Buehler and Pucher 2021).

Of special note are the drivers related to risk minimization, including the ones related to supply chain issues. It is worth noting that even though the relocation decision was taken and implemented before the Covid-19 pandemic, Woom’s top managers were convinced of the benefits of their choice. As noted by one of the respondents: “*Covid-19 has shown how quickly and to what extent entire sectors of the economy can be hit by the crisis and entire supply chains collapse”* (IW_1). However, Covid-19 also emerged as an opportunity, as highlighted by one respondent: *“Due to the pandemic and to the climate crisis, the automotive industry cannot continue to be successful without major changes. Suppliers of the automotive industry are now ready to sit down at the table with people like us. They would not have dealt with children's bicycle manufacturers before”* (IW_1).

Finally, two respondents pointed out that the relocation of assembling activities to Europe can positively affect the public image of the company and will add value to the product. In this respect, one interviewee stated: *“Of course, it’s nice for the image, too. To communicate that this is an Austrian company, and the production sites are located in Europe has a clear advantage. ‘Made-in Europe’ helps”* (IW_3).

When specifically analyzing sustainability issues, it becomes apparent that such elements were widely cited (up to four out of seven respondents), even more than the reduction in logistics costs and shorter lead times (three respondents each). However, the role of sustainability issues in the relocation decision-making phase is somewhat more complex to be appraised. While one of the interviewed managers stated, “*it would be a falsification of the facts if I did not admit that the driver here was not sustainability” (IW_4)*, another specified that *“sustainability is part of every business decision. You don't always manage to go the more sustainable way. But we're working to make that happen. And it is the same with the production in Europe” (IW_5).* In other words, even if sustainability issues were not the decision’s main drivers, they indirectly influenced the strategy to partially relocate production to Europe.

Overall, a central role was played by the company’s values and sustainability strategy. Several respondents from different departments mentioned that producing children’s bicycles is coupled with an intrinsic motivation to contribute to a more sustainable economy. More specifically, the product itself is associated with sustainability-related topics such as green mobility and encourages children to use bicycles more frequently. Therefore, as pointed out by one of the respondents, the decision to near-shore*”fits very well with our overall strategy … because the topic of climate change, long-term threat to the Earth, sustainability as a central future issue, and sustainability as a strategic business goal, that this fits very well with our DNA as a brand and complements each other very well”* (IW_1).

Finally, it emerges that sustainability issues impacting the near-shoring decision were almost all related to the environmental pillar. Even if the company pays specific attention to issues such as fair working conditions, these did not dominate the discussion during the decision-making phase. This may be, at least partially, explained by the higher level of trust recognized towards the German partner. In detail, one respondent noted *“we don't have the issues that something is covered up here or something like that, as it is often said in Asia”* (IW_3). More specifically, Woom managers expected that the new partner would respect the sustainability standards, therefore “*you don't have to constantly check everything again”* (IW_3).

While considering barriers specifically evaluated during the decision-making phase, the most relevant is regarding the higher labor cost (up to four times) associated with producing in Poland instead of Asia. One of the interviewed managers noted: “*It also has major challenges. In any case, it cannot be achieved at the price that can be produced in Asia. This can be partially compensated for by less transport and fewer goods transport. But we believe that it can be done and will pay off”* (IW_5). In this respect, specific attention was devoted to defining production process solutions able to lower the final production cost.

### The implementation phase

When analyzing the implementation phase, two main elements are relevant according to the proposed integrated framework, namely the enabling factors and the barriers. As far as the former is concerned, the interviewees stressed the crucial role of collaboration between Woom and its German partner. Technicians from the two companies actively cooperated in setting up and managing the production lines located in the Polish plant. The collaboration was facilitated by an effective and continuous information exchange implemented between the two partners. The cooperation between Woom and its German partner was facilitated by their previous experience with their suppliers and clients. In this respect, Sprick Cycle top managers noted “*as ODM [Original Design Manufacturer] we manufacture in the name and on behalf of our customers. Through our decades of experience in the bicycle industry, we have been able to make a name for ourselves and establish ourselves as a reliable partner to our customers. We manufacture the bicycle according to the wishes of our customers, handle the shipment to end customers as a service and can guarantee a certain level of warehousing*” (https://sprick-cycle.de). At the same time, Woom had been developing great relationship capacities having collaborated for a long time with Asian partners.

Finally, the choice of a German partner reduced the cultural distance (due to the use of the same language, i.e. German) in addition to the geographic one. Consequently, it is expected that the partnership with Sprick Cycle may further develop in the future. Despite the intense and effective collaboration between Woom and its German partner, some criticalities emerged (they will be discussed in the outcomes subsection). However, the reciprocal trust and the reduced cultural distance also allowed both companies to manage any troubles they encountered.

Another enabling factor emerged in the implementation phase with regard to innovation in production technologies. In this respect, one respondent emphasized *“we would not have been able to take this step without innovation. Definitely not”* (IW_1). As an example, interviewees cited the use of automation for spoke production. Based on an expected further increase in bike demand, Woom aims to further implement automation in the production process in the future, also to counterbalance the higher labor costs of the Polish plant.

Regarding sustainability issues, the respondents pointed out that the German partner, as expected, scrupulously implemented standards regarding working conditions. When they implemented audits regarding the working conditions in the Polish plant, no criticalities emerged, due to the stricter social legislation that is generally adopted in European countries compared to Asian ones.

Finally, respondents stated they did not experience any significant barriers during the implementation phase that hindered Woom to reach its relocation aims. This is, at least partially, explained by the strict and effective collaboration between the partners, which allowed them to overcome any operational criticalities, a subject that will be discussed in the next subsection.

### Outcomes

When considering the outcomes of Woom’s near-shoring strategy, several issues arose, both positive and negative. Among the former, the collected data allowed the identification of 13 different positive outcomes (Table 4). Eight of them are consistent with the initial drivers/motivations that induced the company to partially relocate the assembling activity.

**Table 4:** Benefits experienced after implementing the near-shoring decision

Short-term benefits	Number of respondents (up to 7)	Stated as initial motivation
Increased customer satisfaction	4	X
Shorter travel times and easier access to visit the offshored plant	3	
More control over production at the Polish site	3	X
Closer collaboration and communication with the European Partner	3	X
Improved perceived value due to the ‘Made-in’ effect	2	
Shorter lead times	2	X
Adoption of stricter labor standards	2	
Higher operational flexibility	1	X
Lower cultural distance	1	
Adoption of stricter environmental standards	1	X
Implementation of environmental improvement projects at the Polish plant	1	
Reliability of production & supply	1	X
Better organizational climate at Woom	1	

The most cited benefit is related to marketing issues, more specifically the increased customer satisfaction that was shown by the large majority of Woom’s customers. One interviewee emphasized that some of them explicitly admitted *“this [the near-shoring decision] is an additional argument why I [the customer] buy a Woom. Because I know that it is manufactured under humane conditions, under social aspects in an EU country. Yes, even if not in Germany or Austria, but Poland is just around the corner and that's different than if it is produced in Asia, where I, as the final consumer, do not know: Has it really been produced under humane conditions?”* (IW_1). In other words, the relocation of assembling activity to Poland improved the product value as perceived by Woom’s clients. This is confirmed by two other respondents specifically citing the benefits derived from the “made in Europe effect”. More specifically, one of them stated “*there's a lot of very positive feedback that ‘Made in Europe’ is now written on our bikes”* (IW 4). Such a positive perception is also due to the stricter environmental and labor standards adopted by EU companies compared to the Asian ones. It is worth noting that one of the respondents explicitly stated that relocation strategies implemented by European companies are also likely to have an environmentally positive impact at a macro level. not only at the firm level.

Additionally, some interviewees cited several benefits related to supply chain management issues such as the simplified and more effective control over the production process. In this respect, a respondent noted “*of course, we can control the production process in a European facility more easily than in Asia*” (IW_2). This benefit is also coupled with the reduced travel time to visit the partner’s facility and the reduced cultural distance which, in turn, facilitated the collaboration and simplified the communication with the German partner**.** In this respect, several respondents agreed that *“the essential first simplifications [are] that we are simply much more involved in comparison to Asia and can communicate more openly than with Asian plants”* (IW_6).

At the same time, Woom experienced a dramatic reduction of delivery times, that is the period requested for transferring bikes from the production plant to the Woom’s warehouse. While delivery from Asian suppliers required around six weeks, the Polish plant was able to replenish Woom’s depots in only one day. Even though it was explicitly cited by only one of the respondents, it is worth noting the near-shoring decision had a positive impact on the firm’s “organizational climate”, that is the “properties of the work environment, perceived directly or indirectly by the people who live and work in this environment and assumed to influence motivation and behavior” (Litwin and Stringer 1968, 1). More specifically, interviewed people reported that Woom’s employees were positively reacting to the company's decision to relocate production activities in Europe, even if not in Austria. Finally, the strict collaboration between partners induced the Polish one to further implement initiatives aiming to improve its environmental performance (e.g. waste management projects).

When considering the negative outcomes that emerged after the implementation of the near-shoring strategy, seven key issues were identified as relevant (Table 5). First of all, the large majority of the interviewees highlighted that the adoption of a selective near-shoring strategy (regarding only the assembling phase) did not allow Woom to completely eliminate its dependency on Asian suppliers since it still depends on them for the supply of bike components. Consequently, one respondent pointed out *“but for us, that still means that we are always out of stock because the demand is simply so much higher and because we still cannot increase the capacity because we are missing important components … and they have incredibly long lead times”* (IW_5). Moreover, the situation has become even worse during the Covid-19 pandemic since lead times for some components have increased from 90 to 600 days. Finally, it must be considered that such a criticality may persist in the long term since “*a few components will not be able to be manufactured in Europe”* (IW_5). However, Woom’s management aims *“to make … as much as possible of the product's value chain in Europe”* (IW_5). Hence, the decision to establish production in Poland clearly arises as being the first step in an incremental project to implement a region-based production footprint with production and supply as close to the final sales markets as possible.

**Table 5:** Criticalities/Problems experienced after implementing the near-shore decision

Type of challenge/problem	Number of respondents (up to 7)
Upholding dependency on Asian component producers	5
Higher labor costs	3
Quality issues at the Polish production site	3
Need to assure the same product features in Asia and Poland	2
Negative customer reaction	2
Higher complexity of coordination and communication with suppliers	2
Lower technical expertise of the Polish partner and need to invest in training activity	1

Without considering the higher labor costs, already evaluated during the decision-making phase, a second relevant criticality that arose is regarding difficulties in setting up the production process in the Polish plant. During the interviews – held in May and June 2021 while the production activity in the Polish plant was started in January – one of the respondents stated *“five months is not a long time […] We are still fighting for improvements. That is why it is perhaps a little too early and the impression a bit falsified. At the moment we just have too many topics that don't work that way”* (IW_3). In detail, it was pointed out that, among other problems, the quality at the new production site was one aspect with which the company was not completely satisfied, at least at the beginning. However, such problems have been analyzed and managed through continuous and close collaboration between partners’ technicians.

Since the relocation of assembly activities to Poland is still in the development phase, the production of Woom bikes serving the European markets is still partially undertaken by Asian contractors. Therefore, a specific criticality emerged in terms of the alignment of the outputs of the two product lines. In this respect, one of the respondents stated *“Of course, it is then a big challenge because one production would prefer to do it this way, the other production would prefer to do it differently. And then under certain circumstances, a lot of persuasion or a certain change is necessary in order to keep things the same”* (IW_5).

Finally, a quite unexpected criticality emerged in terms of the company’s image. As pointed out earlier, the large majority of Woom’s customers appreciated the relocation strategy implemented by the company. However, it also *“[…] got a shit storm that should not be underestimated”* (IW_5) from a minority of customers who could not accept Woom was not moving production straight to Austria and, more specifically, to the company’s headquarters in Klosterneuburg. In this respect, it seems that some customers were not aware that Woom had offshored the entire production to Asia shortly after its establishment.

While considering sustainability issues, respondents did not refer to any major criticalities arising after the implementation phase.

Since Woom started its production in the Polish plant only in January 2021, it is also useful to analyze the expected long-term outcomes, at least those regarding sustainability issues. In this respect, respondents referred to Woom’s management aiming to implement further efforts to improve its sustainability performance in the near future. For instance, it has been decided to develop plastic-free packaging for its bicycles. This goal will be reached because of the successful collaboration with the new European partner, while it would be impossible with the former Asian ones. One respondent clearly stated, *“in Poland, we can discuss this directly with the producer and also implement it and, above all, test it”* (IW_6), also because of having a similar level of awareness and perceptions regarding sustainability-related issues.

At the same time, some of the interviewed people stated that Woom aims to implement a circular economy project in the future, in order to *“build Cradle to Cradle capable complete bicycles”* (IW_4) and to achieve the disassembly and recycling of these bicycles. To reach such an ambitious goal, the company would develop an innovative production footprint where facilities focused on the disassembling of used bikes are close to the market destination (in order to make the reverse logistics more efficient) and to the plants where the new products will be manufactured. Therefore, the decision to near-shore to Poland emerges as a first relevant step of a more global vision aiming to improve the sustainability performance of Woom.

Finally, it is important to stress that some respondents raised two concerns at the macro level. More specifically, it was pointed out that relocation strategies implemented by European companies might have adverse effects on the host countries’ economies, especially the ones more involved in global value chains, due to previous waves of production offshoring. Among such adverse effects, of specific note is the loss of jobs in the host countries which will not be counterbalanced by their creation in Western ones, since “*every company that goes back to Europe tries as little as possible to create jobs because it is not affordable and an incalculable risk*” (IW_3).

## Discussion

The main aim of this paper was to investigate the impacts (if any) of social and environmental sustainability on near-shoring strategies. In order to reach this research objective, we adopted an integrated framework based on previous studies by Boffelli and Johansson (2020) and Fratocchi and Di Stefano (2019). Therefore, the impacts of sustainability issues were investigated at three different levels of analysis (Decision making, Implementation and Outcomes) with respect to four different issues (Motivations/drivers; Barriers Enabling factors, and Outcomes). In Table 6 the main results that emerged applying the proposed integrated framework are summarized.

**Table 6:**
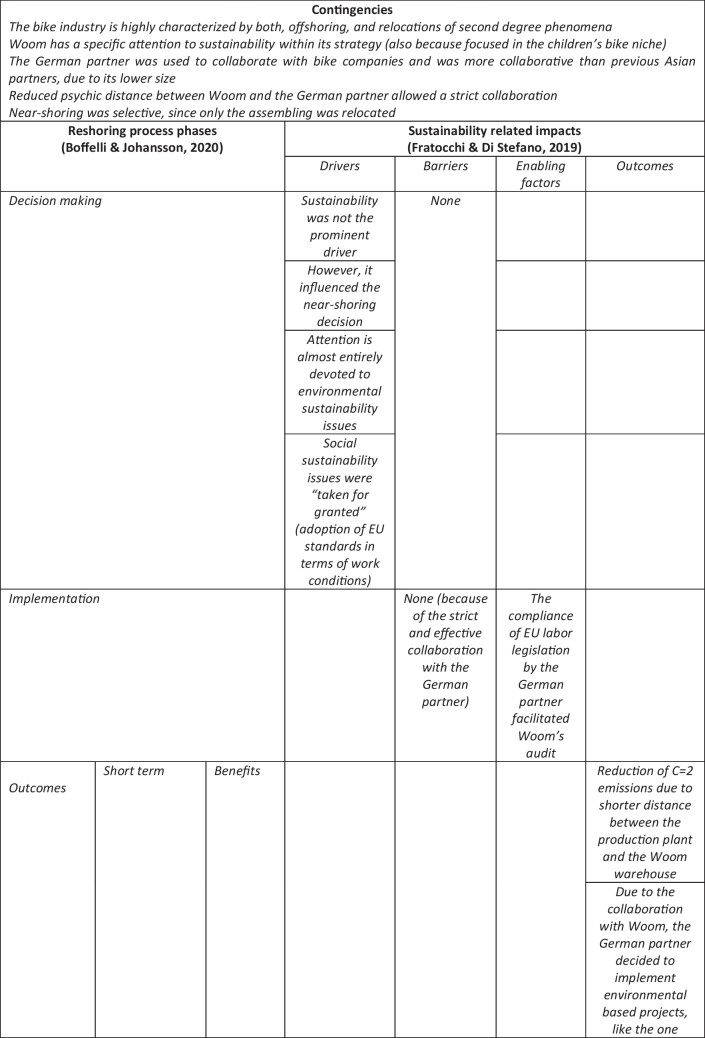

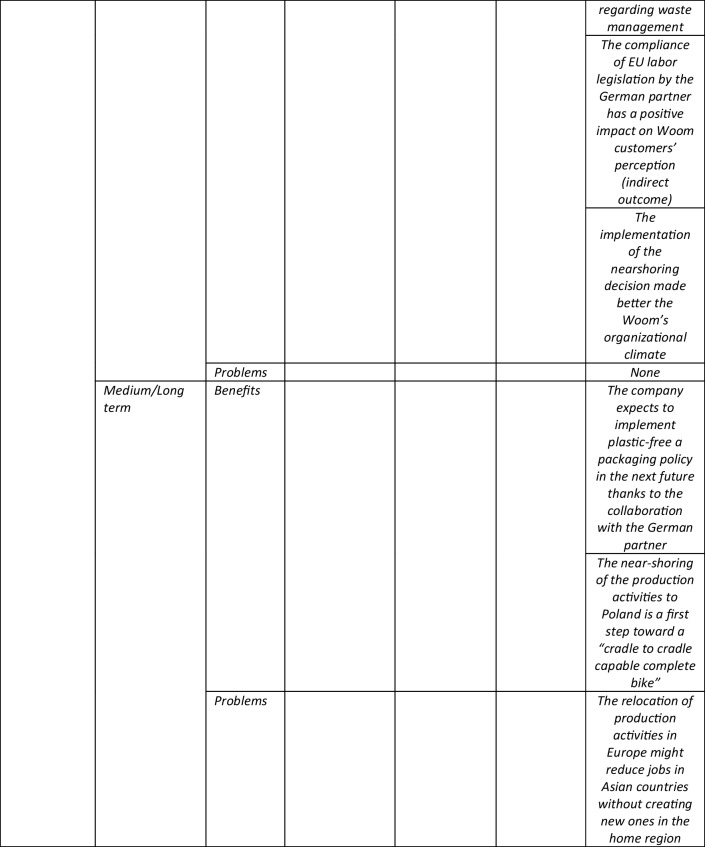
Application of the proposed integrated framework to the Woom case study

Consistent with Fratocchi and Di Stefano (2019), this study shows that environmental and social issues may play, at least partially, different roles when a relocation of second degree decision (like near-shoring) is taken and implemented. However, the proposed integrated framework allows a finer-grained analysis differentiating the impacts of environmental and social sustainability at the different levels of the reshoring process. At the same time, it emerges that the magnitude of such impacts may differ among the analyzed elements (Drivers, Barriers, Enabling factors and Outcomes) and sustainability pillars (environmental vs. social).

Regarding RQ1 (impacts of environmental and social sustainability on the decision making phase), three main results emerged. First, sustainability issues did not represent the main driver/motivation for the Woom’s decision to near-shore its production activity. However, such issues have influenced the company’s relocation decision, given its strategic orientation toward sustainability. In this respect, it is worth noting that such an orientation is increasingly spreading in the bike industry, as confirmed by agreements like the Bike Charta and the Shift Climate Commitment. Such two evidence (the Woom and the industry sustainability orientations) confirm the relevance of internal and external contingencies included in the proposed theoretical framework. When considering the extant reshoring literature, our findings confirm the ones of case studies analyzed by Fratocchi & DI Stefano (2019) and Moradlou et al (2020) where sustainability issues acted as outcomes more than as a motivation/driver.

Secondly, the environmental issues emerged as more relevant than the social ones, confirming previous findings by Fratocchi and Di Stefano (2019) based on the extant reshoring literature. The lower relevance of social sustainability issues in terms of near-shoring drivers, was explained by interviewed people with the assumption that the European Union labor standards are generally higher than the ones required in Asia. Therefore, they are “taken for granted”. This confirms the relevance of national and supra-national legislation when sustainability issues are investigated at the industry and firm levels concerns (Aragòn-Correa et al. 2020). Moreover, it confirms the need to include the analysis of external contingencies (at the host and home country/region) within the proposed theoretical framework.

The third findings regard barriers, which did not emerge related to sustainability in this specific stage of the relocation process. Such evidence might be explained, at least partially, with the specificities of the bike production process, which is generally not highly polluting, compared to chemistry-based processes. Hence, Woom did not experience, e.g., regulatory barriers when wanting to relocate part of the production process to the EU. Moreover, Woom decided to implement a selective near-shoring strategy (Baraldi et al 2019) meaning relocating only the assembling phase, which is traditionally the least polluting. At the same time, the higher European Union labor standards were not perceived as a direct barrier, even if they imply higher labor costs which, in turn, have a relevant effect on the economic sustainability. This may, at least partially, be explained by Woom’s orientation to social and environmental sustainability that contributed to not perceiving stricter legislations as a hindering issue. The scarce relevance of sustainability issues in terms of barriers emerged also when considering RQ2, regarding the implementation phase. This finding was explained by interviewees with the close and effective collaboration developed between Woom and its German partner. Such a collaboration was, at least partially, fostered by the lower psychic distance with respect to Asian contractors. Moreover, it is worth noting that such a collaboration also explicated positive effects in terms of environmental sustainability, like the decision of the German partner to implement a more effective waste treatment process in the Polish plant. This was consistent with the Bike Charta signed by Woom since this agreement explicitly request companies to select sustainable suppliers and establish collaborative actions to increase the sustainability of their products and services This findings is in line with Choudhary et al. (2022) suggestion that production relocation decisions should be coupled with suppliers’ selection processes based on sustainability issues.

A partially different comment arises when considering sustainability issues related to enabling factors. In this respect, audits implemented by Woom in the Polish plant confirmed the adoption of EU labor standards. Such standards emerged as consistent with Woom’s social criteria for contractors’ selection. In contrast, environmental sustainability issues had no impact regarding enabling factors, even if we consider the production technologies. This could be, at least partially, explained by the focus of the relocation strategy on the assembling phase, which generally has no implications in terms of less polluting technologies, in contrast to the case of Martínez-Mora and Merino (2020) with evidence from the jeans industry.

However, the most novel findings – in terms of the previous extant literature on impacts of sustainability on relocations of second degree – concern RQ3 which regards the outcomes level of analysis. More specifically, aside from short-term benefits (respectively, the reduction of CO2 emissions—due to the closeness of the assembling plant to the final market—and the improvement of work conditions due to European Union labor standards), the near-shoring strategy may represent the first step of a long-term strategy which leads to even greater positive impacts, at least in terms of environmental sustainability. In this respect, it is confirmed the usefulness of articulating the outcomes analysis in terms of both, short and medium/long term. More specifically, it emerged that in the medium term, the effective collaboration between Woom and its German partner may generate the reduction of plastics used in the bike packaging. Moreover, the expected relocation of bike components production to Europe will have a further positive impact in terms of CO2 reduction. Finally, when considering the long term, the circular economy project will close the loop, dramatically reducing the company’s carbon footprint. In other words, relocations of second degree may represent an enabling factor for further sustainability strategies.

## Concluding remarks

To the best of our knowledge, this is the first attempt to investigate the influence of sustainability on relocation of second degree addressing the home region, previously investigated case studies having referred to back-shoring (Ashby 2016; Fratocchi and Di Stefano 2019; Martínez-Mora and Merino 2020; Moradlou et al. 2020). This paper contributes to the debate on relocations of second degree in several ways.

First of all, it offers an integrated theoretical framework which allows to evaluate the impacts of two sustainability pillars articulating them according three different levels and four different issues. This enlarge the previous knowledge on the discussed research topic and offer an useful tool for further investigation in other industries.

Secondly, the investigated case study seems to confirm the idea that environmental and social sustainability issues may influence the near-shoring process as already found in the case of back-shoring decisions (Fratocchi and Di Stefano 2019). This would confirm that, at least for sustainability issues, near-shoring and back-shoring strategies share several commonalities, as earlier stated by Ancarani et al. (2015).

Thirdly, our findings suggest some avenues for further research. First of all, we suggest further research should deepen the interdependence between the firm’s relocation strategy and its previous orientation toward sustainability. Our evidence and the previous study by Ashby (2016) induce us to speculate that the relevance of environmental and social issues as a driver is mainly due to the company’s sustainability orientation.

A second issue that needs to be further investigated is related to outcomes and the idea that relocation decisions may activate further strategies aiming to improve the firm’s sustainability performance.

A third avenue for future research is regarding the comparison between impacts of sustainability issues respectively, on back- and near-shoring strategies. Our explorative findings and the previous statement by reshoring scholars (Ancarani et al. 2015) indicate there are no differences, but further investigations are needed.

Our findings also generate useful insights for managers since they confirm the idea that sustainability issues may play various roles when relocation of second degree decisions are taken and implemented. Moreover, our study suggests the idea that the relocation strategy may become an enabling factor for further strategies aimed at improving the firm’s sustainability performances.

Finally, our findings suggest advising policy makers to evaluate the possibility of grading the amount and mix of reshoring aids according to outcomes in terms of environmental and social sustainability. This is consistent with recent suggestions by Elia et al. (2021).

However, the paper is not without limitations; the biggest is its focus on a single case study, albeit such a methodology is consistent with the explorative aims of our research. Future studies should consider other cases belonging to different industries and home countries/regions. With respect to the latter issue (home region), of special note is the role of environmental and social legislation. While in the Woom case study, the Austrian company assumed that the German partner would adopt the EU working conditions standards, in the case of near-shoring of US companies in Mexico such an assumption cannot be made. Lastly, it has to be considered that this research was conducted shortly after the case company finished implementing their nearshoring decision. Therefore, only the short-term outcomes could be analyzed and, therefore, it is recommended to further analyze sustainability-related long-term outcomes of reshoring decisions to gain a thorough understanding of the variety of different outcomes over time.

## Appendix A

### Interview Guide

Company details:

1. Can you give me briefly some information about the company?
2. What is your position in the company?

(I) Offshoring:
  1. Can you tell me a bit about the offshoring process?
    - What activities were offshored?
    - When were production activities initially offshored?
    - How long were these activities offshored?
    - To which countries where these activities offshored?
    - Did you contract suppliers?
  2. What events lead to offshoring production activities to XYZ?

(II) Reshoring: Decision-making
  1. Why did the company decide to reshore these activities?
    - Were specific events contributing to reshoring the production activities to XYZ?
  2. How was the decision made to reshore?
  3. What aspects were taken into consideration when making this decision?
    - Were environmental aspects playing a role?
      (i) And if so, which?
      (ii) Why were these aspects playing a role in the decision making process?
      (iii) What role did these aspects play in the decision making process?
    - Were social aspects playing a role?
      (i) And if so, which?
      (ii) Why were these aspects playing a role in the decision making process?
      (iii) What role did these aspects play in the decision making process?

(III) Reshoring: Implementation process
  1. Can you tell me about the implementation process of moving production activities?
    - What steps did you take?
    - Were the production activities insourced or outsourced in the home country?
    - Who participated in the implementation process?
  2. What aspects were taken into consideration during the implementation process?
    - Did environmental aspects play a role during the implementation?
      (i) And if so, which?
      (ii) Why did you take these into consideration?
      (iii) What role did these aspects play in the implementation process?
    - Did social aspects play a role during the implementation?
      (i) And if so, which?
      (ii) Why did you take these into consideration?
      (iii) What role did these aspects play in the implementation process?
    - Did innovation play a role during the implementation?

(IV) Reshoring: Outcomes
  1. What were the immediate short-term effects after reshoring on firm-level?
    - Did any unexpected effects occur?
    - Did any unforeseen challenges arise as a short-term consequence of reshoring?
  2. What are the long-term effects on firm level? So what are the outcomes now?
    - Did any unexpected effects occur?
    - Sustainability connection
  3. Did the reshoring process have effects on the environmental performance of the company?
    - If so, which aspects have changed?
  4. Did the reshoring process have effects on the social performance of the company?
    - If so, which aspects have changed?
  5. Was the company confronted with any difficulties or problems during the process?

Closing

1. Are there any questions you would like to come back to, or want to revise your answer?
2. Do you have any other comments or issues you want to share?
